# Self-Reported quality of life in adults with attention-deficit/hyperactivity disorder and executive function impairment treated with lisdexamfetamine dimesylate: a randomized, double-blind, multicenter, placebo-controlled, parallel-group study

**DOI:** 10.1186/1471-244X-13-253

**Published:** 2013-10-09

**Authors:** Lenard A Adler, Bryan Dirks, Patrick Deas, Aparna Raychaudhuri, Matthew Dauphin, Keith Saylor, Richard Weisler

**Affiliations:** 1 Department of Psychiatry and Child and Adolescent Psychiatry, New York University School of Medicine and Psychiatry Service, New York VA Harbor Healthcare System, New York, NY, USA; 2 Clinical Development and Medical Affairs, Shire Development LLC, Wayne, PA, USA; 3 NeuroScience, Inc., Herndon, VA, USA; 4 Duke University Medical Center, Durham, NC, USA; 5 University of North Carolina at Chapel Hill, Chapel Hill, NC, USA

**Keywords:** Lisdexamfetamine dimesylate, LDX, ADHD, Quality of life, Executive function

## Abstract

**Background:**

This study examined the effects of lisdexamfetamine dimesylate (LDX) on quality of life (QOL) in adults with attention-deficit/hyperactivity disorder (ADHD) and clinically significant executive function deficits (EFD).

**Methods:**

This report highlights QOL findings from a 10-week randomized placebo-controlled trial of LDX (30–70 mg/d) in adults (18–55 years) with ADHD and EFD (Behavior Rating Inventory of EF-Adult, Global Executive Composite [BRIEF-A GEC] ≥65). The primary efficacy measure was the self-reported BRIEF-A; a key secondary measure was self-reported QOL on the Adult ADHD Impact Module (AIM-A). The clinician-completed ADHD Rating Scale version IV (ADHD-RS-IV) with adult prompts and Clinical Global Impressions-Severity (CGI-S) were also employed. The Adult ADHD QoL (AAQoL) was added while the study was in progress. A post hoc analysis examined the subgroup having evaluable results from both AIM-A and AAQoL.

**Results:**

Of 161 randomized (placebo, 81; LDX, 80), 159 were included in the safety population. LDX improved AIM-A multi-item domain scores versus placebo; LS mean difference for Performance and Daily Functioning was 21.6 (ES, 0.93, *P*<.0001); Impact of Symptoms: Daily Interference was 14.9 (ES, 0.62, *P*<.0001); Impact of Symptoms: Bother/Concern was 13.5 (ES, 0.57, *P*=.0003); Relationships/Communication was 7.8 (ES, 0.31, *P*=.0302); Living With ADHD was 9.1 (ES, 0.79, *P*<.0001); and General Well-Being was 10.8 (ES, 0.70, *P*<.0001). AAQoL LS mean difference for total score was 21.0; for subscale: Life Productivity was 21.0; Psychological Health was 12.1; Life Outlook was 12.5; and Relationships was 7.3. In a post hoc analysis of participants with both AIM-A and AAQoL scores, AIM-A multi-item subgroup analysis scores numerically improved with LDX, with smaller difference for Impact of Symptoms: Daily Interference. The safety profile of LDX was consistent with amphetamine use in previous studies.

**Conclusions:**

Overall, adults with ADHD/EFD exhibited self-reported improvement on QOL, using the AIM-A and AAQoL scales in line with medium/large ES; these improvements were paralleled by improvements in EF and ADHD symptoms. The safety profile of LDX was similar to previous studies.

**Trial registration:**

ClinicalTrials.gov, NCT01101022

## Background

Attention-deficit/hyperactivity disorder (ADHD) is a neurobehavioral disorder that often persists into adulthood. The estimated prevalence of ADHD in adults is 4.4% [1]. It is well-recognized that ADHD manifests not only as impairments in the core ADHD symptoms (inattention, hyperactivity, impulsivity), but is also correlated with executive function deficits (EFD; impairment in cognitive self-regulatory processes) [2-4] that impact goal-directed behavior and control emotional functioning [5,6]. Executive function (EF) domains include verbal and nonverbal working memory, emotional self-regulation, shifting of attention or focus, and planning and problem solving among others [7,8]. Deficits in EF remain stable even in adulthood [9,10], although presentation of core ADHD symptoms may change with age. EFD affects outcomes in education, work, social relationships, and psychosocial functioning [11,12].

Validated rating scales of behaviors thought to be associated with EF include the self-reported Brown Attention-Deficit Disorder Scale (BADDS) [13], Barkley Deficits in Executive Functioning Scale [14], and Behavior Rating Inventory of Executive Function-Adult Version (BRIEF-A) [15]. The BRIEF-A scoring employs T-scores, where the normative population mean is set at 50, with a SD of 10. A T-score ≥65 indicates clinically significant EF impairments [15] of complex behavioral tasks in daily real-world settings [2,5,8,16]. Compared with neuropsychological tests of EF, behavioral assessments may be better predictors of impairment in major life activities [17]. Such impairments may be reflected in quality of life (QOL), a subjective perception of well-being in various functional life domains including physical, psychological, cognitive, and social [18]. Since deficits in EF domains may impact QOL across various life domains, concurrent assessments of EF and QOL may more fully quantify patient improvement on ADHD treatment.

Adults with symptoms of ADHD may display impairments in executive functioning and in QOL [19]. Moreover, improvements in ADHD symptoms may coincide with improvements in both EF and QOL [20]. Instruments assessing QOL may be disease-specific or nonspecific. In either case, these may enable the physician to assess clinical treatment outcomes that may be perceived as important from a patient perspective and, hence, perceived as an appropriate means to monitor treatment progress [21].

The self-reported Adult ADHD Impact Module (AIM-A) [22] multi-item scale is a validated QOL measure that has demonstrated treatment sensitivity in a double-blind, placebo-controlled study of a long-acting psychostimulant [23]. Another validated QOL measure, the 29-item self-reported Adult ADHD QoL (AAQoL) scale [24], exhibited responsiveness to ADHD treatment in an 8-week, randomized, placebo-controlled trial of the nonstimulant atomoxetine [21].

The long-acting prodrug psychostimulant lisdexamfetamine dimesylate (LDX) is indicated in the United States for ADHD in children (6 to 12 years), adolescents (13 to 17 years), and adults [25]. LDX has demonstrated efficacy in reducing core ADHD symptoms in a randomized, controlled trial in adults [26], using the ADHD Rating Scale IV (ADHD-RS-IV) [27,28]; and was associated with improvements from baseline in EF, using the validated, self-reported BADDS [13,29], during the open-label phase of a modified analog classroom study (4-week) of adults with ADHD [30]. In the modified analog classroom study, LDX treatment demonstrated efficacy in adults with ADHD who had significant impairments in ADHD core symptoms and EF. Moreover, LDX demonstrated efficacy in QOL as assessed by AIM-A [31].

Further randomized, double-blind, placebo-controlled studies may be useful to confirm the impact of psychostimulant treatment on QOL domains in adults with ADHD and EFD, and provide clinicians with information on the types of assessment tools available as well as their use in making patient-relevant assessments. A randomized, controlled trial using LDX in adult participants with both ADHD and EFD reported improvement in EFD with BRIEF-A Global Executive Composite (GEC) LS mean (SE) change from baseline of -11.1 (1.72) for placebo and -22.3 (1.67) for LDX, respectively (primary efficacy measure, *P*<.0001; effect size of 0.74) [32]. During the course of the study, 2 separate QOL instruments were used as secondary outcomes. With these data, we may be able to explore ADHD treatment effects, not only on core symptoms (inattention, hyperactivity, and impulsivity), but also on EFD and self-reported QOL during the course of that treatment.

### Objectives

This report focuses on the secondary study outcomes reported including the effects of LDX treatment on participant-perceived QOL measures, the AIM-A, and the AAQoL. The AAQoL was added to the study as a protocol amendment and was not used on all participants. Also reported in this study is a post hoc subgroup analysis of those participants with both AIM-A and AAQoL scores at baseline. The primary objective of the present study was to demonstrate the efficacy of LDX versus placebo in improving EF in participants with ADHD and coexisting EFD. The primary and other secondary endpoints of this study have been presented in detail elsewhere [32].

## Methods

### Study design

Detailed description of study methodology and primary results for this randomized, double-blind, multicenter, placebo-controlled, parallel-group efficacy and safety study of LDX versus placebo have been previously reported [32]. Enrolled eligible adults (aged 18 to 55 years inclusive at time of consent) met full *Diagnostic and Statistical Manual*, *Fourth Edition*, *Text Revision* (DSM-IV-TR) [33] criteria for a diagnosis of ADHD and had clinically significant EFD [32]. Herein, we report on QOL measures.

The study protocol was approved by the independent Institutional Review Board, Copernicus Group Independent Review Board (Research Triangle Park, NC). The study was conducted in accordance with the International Conference on Harmonisation Guideline for Good Clinical Practice E6 [34]. Following detailed explanation of the study, all adult participants and adult informants provided written informed consent.

Key inclusion criteria were adults with a reported baseline ADHD-RS-IV total score of ≥28 and significant EFD, assessed using a self-reported BRIEF-A GEC T-score of ≥65 at baseline. Participants were required to be in a close domicile relationship for at least 6 months prior to screening with an individual capable of participating as an informant on the participant’s symptoms and behaviors in multiple social settings. Key exclusion criteria included adults who exhibited comorbid psychiatric conditions, controlled or uncontrolled, including severe axis I or II disorders, cardiovascular disease, a history of moderate to severe hypertension, current ADHD therapy, and a history of failure to respond to amphetamine therapy. After screening and washout for up to 4 weeks, eligible participants meeting inclusion and exclusion criteria were randomized in a 1:1 ratio at baseline (week 0) to placebo (n=81) or optimized LDX 30, 50, or 70 mg/d (n=80) during a 10-week, double-blind treatment period. Randomization was stratified by cohabitation status (married/cohabitating, not married/cohabitating); within-strata participants were randomized to placebo or LDX groups based on a 4-digit randomization code assigned once eligibility was established. Blinded assignment of treatment groups was accomplished through an interactive voice/web response system. During the 4-week dose-optimization period, treatment (morning; 7 am ±2 hours) was initiated at 30 mg/d and titrated in 20-mg/week increments to an optimal dose (30, 50 or 70 mg/d, as tolerated). The optimal dose was based on a baseline ADHD-RS–IV with adult prompts total score reduction of ≥30% and a CGI-Improvement (CGI-I) [35] scale rating of very much improved or much improved. Participants were continued at their optimal dose with no further dose adjustments (increase or decrease) permitted during the 6-week dose-maintenance period. The final dose-maintenance period visit was at week 10, with participants who did not complete the study assessed at an early termination (ET) visit.

### Efficacy measures

#### ***BRIEF-A***

The self-reported BRIEF-A consists of 75 items that are scored as occurring never, sometimes, or often based on behavior in the 3 weeks prior to assessment [15]. The EF domains assessed included the ability to initiate behaviors, inhibit competing actions or responses to stimuli, plan and organize when solving complex problems, shift problem-solving strategies easily, regulate emotions, monitor and evaluate behavior, and use working memory efficiently.

#### ***ADHD-RS-IV***

The clinician-completed ADHD-RS-IV with adult prompts total score evaluated change from baseline in ADHD core symptoms at week 10/ET with LDX versus placebo. The ADHD-RS-IV with adult prompts consists of 18 symptom items (inattention, hyperactivity, and impulsivity), based on current DSM-IV-TR criteria for ADHD; items are scored on a 4-point scale, ranging from 0 (no symptoms) to 3 (severe symptoms); total scores range from 0 to 54.

#### ***AIM-A***

The self-reported, validated AIM-A (key secondary efficacy measure) evaluated QOL with LDX treatment in comparison with placebo. The AIM-A consists of 6 multi-item global domain scales. For this study, 4 were considered key secondary efficacy measures: Performance and Daily Functioning, Impact of Symptoms: Daily Interference, Impact of Symptoms: Bother/Concern, and Relationships/Communication. The other 2 were additional, but not key, secondary efficacy measures: living with ADHD, and general well-being. The recall period for the multi-item scales is over 1 week and responses are based on a Likert scale that ranges from 1 to 5. Raw scores were transformed to a 0 to 100 scale with higher scores indicating a better QOL. The AIM-A also contains overall QOL questions 1 to 4:

1. “On a scale of 1–10 how would you rate the overall quality of your life right now?” (higher scores representing more positive ratings).

2. “Has ADHD and its symptoms limited your ability to achieve what you want in life?” was rated by participants on a scale of 1 (yes, a lot) to 4 (no, not at all) with lower scores representing more limitation.

3. “Do you feel you are on the right track with your life?” was participant-rated on a scale of 1 (yes, definitely) to 3 (no, not at all).

4. “How much do you agree with the statement: Over the past few weeks, I’ve had more good days than bad days” was scored on a scale of 1 (strong agreement) to 5 (strong disagreement).

The AIM-A was assessed at baseline (dose-optimization phase) and at week 4 and week 10/ET (dose-maintenance phase).

#### ***AAQoL***

The self-reported AAQoL is a validated 29-item scale consisting of a total score and 4 subscales (life productivity, psychological health, life outlook, and relationships) designed to assess health-related QOL in adults with ADHD. Items were scored on a 5-point Likert scale ranging from 1 (not at all/never) to 5 (extremely/very often). Raw scores were transformed to a 0 to 100 scale with higher scores indicating a better QOL. The AAQoL was evaluated at baseline (dose-optimization phase) and at week 4 and week 10/ET (dose-maintenance phase). The self-reported AAQoL was included after protocol amendment 1 (July 8, 2010) that deleted the Sheehan Disability Scale; hence not all study participants completed the AAQoL scale. Only new participants, who performed the assessment at baseline, were able to complete the AAQoL.

#### ***Post hoc analyses***

A post hoc subgroup analysis of the AIM-A (key secondary) scale for participants enrolled with both baseline AIM-A and AAQoL scores was conducted.

### Safety measures

Safety assessments included treatment-emergent adverse events (TEAEs), vital signs (systolic [SBP] and diastolic blood pressure [DBP]), and electrocardiogram (ECG).

### Statistical analyses

All efficacy assessments presented were for the full analysis set (FAS), which included all participants who took 1 treatment dose in the double-blind evaluation phase and had 1 postbaseline/randomization primary efficacy assessment. Placebo and LDX groups were compared using an analysis of covariance (ANCOVA) for change, with treatment groups as factor and baseline as the covariate. Effect size is the (mean change from baseline in LDX group minus mean change from baseline in placebo group) divided by pooled standard deviation (SD). For the post hoc analysis, there were no multiplicity adjustments performed on the subgroup statistical comparisons. Safety data were reported on the set of all participants who took at least 1 treatment dose in the double-blind phase of the study.

Sample size was determined assuming a treatment effect size of 0.56 by a 2-sided, 2-sample t-test at the significance level of 0.05 requiring at least 52 participants in each treatment group to provide 80% power. The sample size was adjusted up to 80 participants per group to account for key secondary endpoints and anticipated early dropouts.

A sequential, hierarchical hypothesis testing of the multiple efficacy endpoints was prespecified to maintain control of Type I errors based on multiple hypothesis testing. When a nonsignificant difference was first observed at a *P*>.05 level, no further testing of the remaining hypotheses was allowed. There was no hypothesis testing or inferential assessment conducted for the remaining endpoints and the corresponding null hypotheses were not rejected.

## Results

Demographic data and participant disposition were previously reported in detail [32]. Of 161 adults enrolled, the safety population included 159 participants, with 80 participants receiving at least 1 dose of placebo and 79 receiving at least 1 dose of LDX. Overall, baseline and demographic characteristics for placebo and LDX groups were comparable. The majority of participants was white (85.5%) and non-Hispanic/non-Latino (92.5%). The FAS included 154 participants (75 placebo and 79 LDX). There were 26 placebo participants and 28 LDX participants who had both AIM-A and AAQoL baseline measures and who were included in the post hoc comparison of these 2 measures.

### Efficacy

#### ***Self-reported BRIEF-A GEC, ADHD-RS-IV, and CGI-S***

At baseline, the mean (SD) BRIEF-A GEC T-score was 79.4 (8.68) for placebo and 79.5 (8.01) for LDX. Detailed results for self-reported BRIEF-A GEC were previously described [32]. At baseline, participants had a mean (SD) ADHD-RS-IV total score of 39.9 (6.83) for placebo and 39.9 (7.37) for LDX, respectively, in the FAS. At week 10/ET, LS mean (SE) changes in the ADHD-RS-IV total score were -10.3 (1.38) for placebo and -21.4 (1.35) for LDX (*P*<.0001; effect size of 0.94). At baseline on the CGI-S, all participants were rated moderately to severely ill; whereas at week 10/ET, 22.7% of placebo and 51.9% of LDX participants were classified as not at all or borderline mentally ill.

#### ***AIM-A multi-item global domain scales***

**Figure 1:**
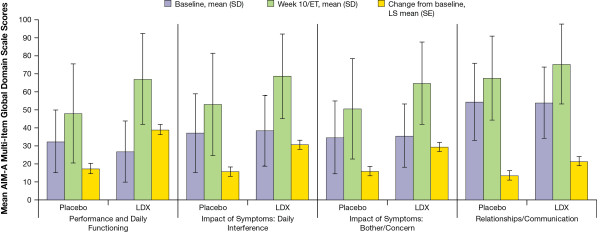
Self-reported mean AIM-A multi-item global domain scores.

Treatment with LDX resulted in greater improvement compared with placebo on all AIM-A global multi-item domain scales at week 10/ET (*P*≤.0302 for all). The key secondary efficacy measures are shown in Figure 1. On the Performance and Daily Functioning Scale, LS mean (SE) change was 17.2 (2.91) for placebo and 38.8 (2.84) for LDX, with an LS mean difference (95% CI) of 21.6 (13.5, 29.7) and an effect size of 0.93. For the Impact of Symptoms: Daily Interference Scale, the LS mean (SE) change was 15.7 (2.58) for placebo and 30.6 (2.52) for LDX and the LS mean difference (95% CI) was 14.9 (7.8, 22.0), with the effect size of 0.62. For the Impact of Symptoms: Bother/Concern scale, LS mean (SE) change score was 15.8 (2.60) for placebo and 29.3 (2.53) for LDX and the LS mean difference (95% CI) was 13.5 (6.3, 20.7), with an effect size 0.57. For the Relationships/Communication scale score the LS mean (SE) change score was 13.4 (2.56) for placebo and 21.2 (2.49) for LDX, with an LS mean difference (95% CI) of 7.8 (0.8, 14.9) and an effect size of 0.31.

**Figure 2:**
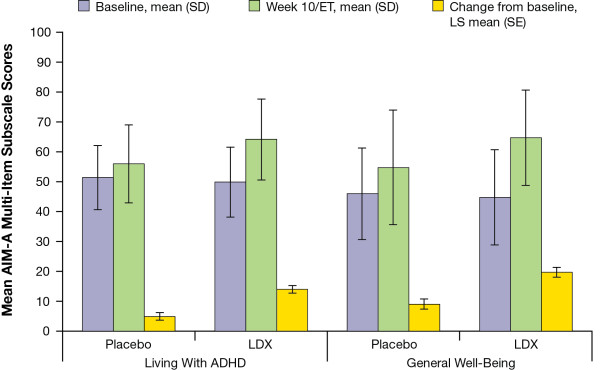
**Mean self-reported AIM-A multi**-**item subscale scores.**

On the other domain subscales (Figure 2), the Living With ADHD subscale had an LS mean (SE) change from baseline of 4.9 (1.33) for placebo and 14.0 (1.30) for LDX, LS mean difference (95% CI) of 9.1 (5.4, 12.7) (*P*<.0001), effect size of 0.79; and the General Well-Being subscale had an LS mean (SE) change from baseline of 9.0 (1.73) for placebo and 19.7 (1.69) for LDX, LS mean difference (95% CI) of 10.8 (6.0, 15.5) (*P*<.0001), effect size of 0.70.

#### ***AIM-A overall QOL questions 1 and 4***

At baseline, the mean (SD) score on question 1 was 5.7 (2.00) and 5.7 (1.71) for placebo and LDX, respectively. The LS mean (SE) change score at week 10/ET for question 1 was 1.0 (0.16) and 1.6 (0.16) for placebo and LDX, respectively. For this question, the LS mean difference (95% CI) was 0.5 (0.1, 1.0; *P*=.0184), with the effect size of 0.29. For AIM-A Overall QOL question 4 the mean (SD) score at baseline for placebo and LDX, respectively, was 2.9 (1.17) and 2.9 (1.09), and the LS mean (SE) change scores at week 10/ET were -0.4 (0.11) and -1.0 (0.11). The LS mean difference (95% CI) was -0.6 (-0.9, –0.3) for Question 4 (*P*=.0004) and the effect size was 0.44.

#### ***AIM-A overall QOL questions 2 and 3***

For AIM-A overall QOL questions 2 and 3, there was no hypothesis testing or inferential assessment conducted based on the sequential hierarchal hypothesis testing procedure used in this study. For AIM-A overall question 2 (Table 1), at baseline, about 52.0% and 51.9% of the participants in the placebo and LDX treatment groups, respectively, responded that their ADHD symptoms limited “a lot” of their ability to achieve and another 37.3% and 36.7% in the placebo and LDX groups, respectively, responded “yes, some” limitation. At week 10/ET, there was a shift to improvement in both groups with 62% to 64% reporting “a lot” or “some” limitation and the proportions reporting little or no limitations in both groups increasing from 10%–11% to 36%–37.9%. For AIM-A overall question 3 (Table 1), at baseline, more than 60% of participants in each treatment group considered themselves “somewhat” on the right track, and 20% of each group answered “not at all.” At week 10/ET, there was a shift to improvement with 20% of participants in the placebo group and 39.2% in the LDX group reporting “yes, definitely,” and 69.3% on placebo and 54.4% on LDX responding “yes, somewhat” on the right track.

**Table 1:** **AIM**-**A overall questions 2 and 3 percent response at baseline and week 10**/**ET**

		**Placebo (n=75)**		**LDX (n=79)**	
**AIM-A Overall QOL Question**	**n (%)**	**Baseline**	**Week 10/ET**	**Baseline**	**Week 10/ET**
Question 2: Has ADHD and its symptoms limited your ability to achieve what you want in life?	Yes, a lot	39 (52.0)	31 (41.3)	41 (51.9)	24 (30.4)
	Yes, some	28 (37.3)	17 (22.7)	29 (36.7)	25 (31.6)
	Yes, a little	7 (9.3)	23 (30.7)	8 (10.1)	22 (27.8)
	No, not at all	1 (1.3)	4 (5.3)	1 (1.3)	8 (10.1)
Question 3: Do you feel you are on the right track with your life?	Yes, definitely	9 (12.0)	15 (20.0)	12 (15.2)	31 (39.2)
	Yes, somewhat	51 (68.0)	52 (69.3)	51 (64.6)	43 (54.4)
	No, not at all	15 (20.0)	8 (10.7)	16 (20.3)	5 (6.3)

#### ***Self-reported AAQoL scale***

**Figure 3:**
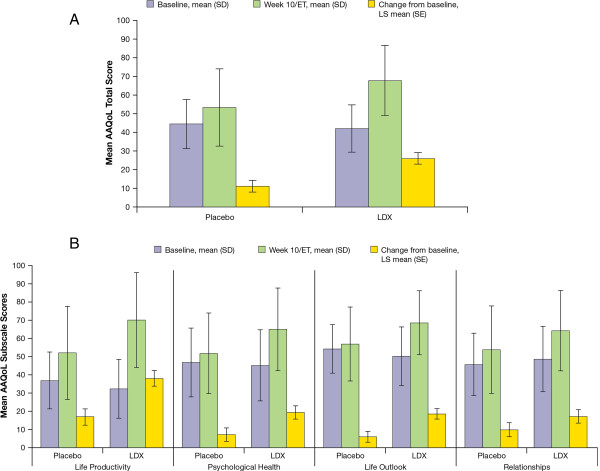
**Change from baseline at week 10/ET in AAQoL A) total and B) subscale scores.**

For the AAQoL, no hypothesis testing or inferential assessments were conducted based on the sequential hierarchal hypothesis testing procedure used in this study. At week 10/ET, the self-reported AAQoL total score change was numerically greater compared with placebo for LDX participants (Figure 3A); the LS mean difference (95% CI) was 14.7 (5.9, 23.6). Changes from baseline scores were numerically improved with LDX compared to placebo for all AAQoL subscales, Life Productivity, Psychological Health, Life Outlook, and Relationships (Figure 3B). The LS mean differences (95% CI) at week 10/ET were 21.0 (8.4, 33.6) for Life Productivity; 12.1 (1.6, 22.5) for Psychological Health; 12.5 (4.2, 20.8) for Life Outlook; and 7.3 (-3.4, 18.0) for Relationships subscales.

### Post hoc analyses

#### ***Post hoc AIM-A multi-item subgroup analyses***

For participants enrolled with baseline self-reported AIM-A and AAQoL scores (n=26 placebo; n=28 LDX), all post hoc subgroup AIM-A multi-item scales were numerically improved from baseline for LDX versus placebo at week 10/ET and were similar in magnitude to FAS results. On the Performance and Daily Functioning scale, LS mean (SE) change was 17.4 (4.95) for placebo and 37.7 (4.77) for LDX, with an LS mean difference (95% CI) of 20.3 (6.5, 34.2). For the Impact of Symptoms: Daily Interference scale, the LS mean (SE) change was 20.1 (4.54) for placebo and 31.8 (4.38) for LDX, and the LS mean difference (95% CI) was 11.7 (-1.0, 24.3). For the Impact of Symptoms: Bother/Concern scale, LS mean (SE) change score was 19.1 (4.37) for placebo and 33.5 (4.21) for LDX, and the LS mean difference (95% CI) was 14.4 (2.2, 26.6). For the Relationships/Communication scale, the LS mean (SE) change was 11.5 (3.49) for placebo and 24.9 (3.36) for LDX, with an LS mean difference (95% CI) of 13.5 (3.7, 23.2). The Living With ADHD subscale LS mean (SE) change from baseline was 6.5 (1.78) for placebo and 14.2 (1.72) for LDX, with an LS mean difference (95% CI) of 7.7 (2.7, 12.7). On the General Well-Being subscale, the LS mean (SE) change from baseline was 8.3 (2.40) for placebo and 17.2 (2.31) for LDX, with an LS mean difference (95% CI) of 8.9 (2.2, 15.6).

Responses to AIM-A, question 1: “On a scale of 1–10 how would you rate the overall quality of your life right now?” and question 4: “How much do you agree with the statement: Over the past few weeks, I’ve had more good days than bad days,” were numerically but not statistically improved versus placebo with LDX at week 10/ET. The LS mean (SE) change at week 10/ET for placebo and LDX, respectively, were 0.8 (0.31) and 1.4 (0.30) for question 1, and -0.5 (0.22) and -1.0 (0.21) for question 4.

### Safety measures

TEAEs were reported by 47 of 80 (58.8%) placebo participants and 62 of 79 (78.5%) LDX participants. TEAEs and other safety findings are presented in detail elsewhere [32]. For placebo and LDX, respectively, TEAEs with a frequency ≥10% in either group were decreased appetite (6.3% and 32.9%), dry mouth (7.5% and 31.6%), headache (2.5% and 25.3%), feeling jittery (0% and 12.7%), insomnia (3.8% and 12.7%), initial insomnia (6.3% and 10.1%), irritability (3.8% and 10.1%), and decreased weight (0% and 10.1%). There were no deaths or serious AEs during the trial, and most participants reported TEAEs that were mild or moderate in severity. Severe TEAEs were reported in 3 (3.8%) LDX and 3 (3.8%) placebo participants. Discontinuations due to TEAEs included 2 (2.5%) placebo participants and 5 (6.3%) LDX participants.

Mean changes in vital signs were not clinically meaningful. Respectively, among placebo and LDX groups at week 10/ET, mean (SD) increases from baseline in: SBP were 1.7 (9.22) and 2.6 (8.39) (mm Hg); DBP were 1.5 (8.85) and 1.7 (7.60) (mm Hg); and pulse rate were 3.3 (8.35) and 5.4 (10.79) (bpm). There were no changes in mean laboratory parameters over time that were deemed to be of clinical concern.

## Discussion

This population of adults with ADHD and significant EF impairment at baseline demonstrated significantly improved QOL versus placebo as assessed by the self-reported AIM-A in the subscales of Performance and Daily Functioning, Impact of Symptoms: Daily Interference, Impact of Symptoms: Bother/Concern, and Relationships/Communications. Moreover, self-reported responses to AIM-A questions 1 to 4 suggested that participants perceived better overall improvement in QOL with LDX treatment versus placebo. These data indicate that with LDX treatment, adults who have ADHD and EFD showed significant improvement on the self-reported AIM-A QOL. Numerical improvements over placebo were also seen with LDX treatment on the self-reported AAQoL total and subscale scores, although the prespecified testing hierarchy precludes inferential statistical analysis of the AAQoL. The self-reported QOL data in this analysis suggest comparable findings using either QOL measure (AIM-A or AAQoL).

The ability to be productive in the workplace is an important issue for adults with ADHD in an increasingly competitive work environment [36-38]. The study by Kupper et al. [38], which reviewed published literature reporting on the impact of ADHD on work productivity and occupational health, suggested that adults with ADHD exhibited decreased work performance and productivity, increased behavioral issues (irritability and frustration), absenteeism, increased workplace accidents or injury, as well as increased indirect effects (eg, substance use and criminality) on occupational health. Real-life outcomes, such as productivity, can be assessed using these validated QOL scales and can be important for clinicians to determine ADHD treatment response. Adults with ADHD in this study reported significantly improved QOL with LDX treatment compared to placebo on the AIM-A Performance and Daily Functioning subscale and numerical improvement on the AAQoL Life Productivity domain.

The AIM-A QOL data from the present study were consistent with a 7-week, randomized controlled ADHD study of an extended-release mixed amphetamine salts formulation that also demonstrated significantly improved QOL at study endpoint relative to placebo [39]. These study data suggest that significant improvement in QOL in those with ADHD may become apparent in as early as 7 weeks after initiating psychostimulant treatment.

Another 14-week randomized controlled study [40] demonstrated that the AAQoL total score was significantly improved from baseline versus placebo with atomoxetine (a nonstimulant), with an effect size of 0.24. In that study, however, significant improvement with atomoxetine versus placebo was only demonstrated on 1 of the 4 subscales, Psychological Health. Another 6-month, double-blind study, using the AAQoL scale, showed greater numerical improvement with atomoxetine than placebo in adult participants with ADHD of at least moderate severity, with the Life Outlook subscale score showing significant improvement versus placebo [19].

In the current study, QOL measures improved along with improvements in self-reported EF, using the BRIEF-A, and investigator-reported symptom improvement, using the ADHD-RS-IV with adult prompts. As described in the primary study [32], LDX treatment versus placebo significantly improved deficits in EF (BRIEF-A GEC T-scores of ≥65) bringing scores well within the normative range. Likewise, significant improvements in ADHD symptoms with LDX treatment compared to placebo were also demonstrated with ADHD-RS-IV and CGI-I scores. Overall, these findings suggest that LDX treatment might result in improvements in multiple functional areas affected by ADHD, and that researchers as well as clinicians have available tools and resources to monitor such improvements [41].

### Limitations

Since adults with comorbid psychiatric disease and significant cardiovascular disease were excluded from the current study population, caution should be taken when generalizing results to the overall population. In addition, study participants were required to meet a prespecified level of EFD, and this further limits generalization. The subjective, participant-reporting nature of such rating scales should also be considered because it is not possible to account for variability in rating scores among individual participants. Also, the short duration of the trial limits the ability to specify the long-term effects of LDX on assessments of QOL. The predefined hierarchical statistical testing program precludes statistical inferences on some of the measures, and no statistical inferences were made based on the post hoc analyses.

## Conclusions

Adults with ADHD and clinically significant real-life EFD exhibited self-reported improvement on all QOL subscales and the majority of domains, using the self-reported AIM-A and AAQoL scales, respectively. The effects on QOL paralleled improvements in self-reported EF ratings and ADHD symptoms, as reported elsewhere [32]. Effect sizes were mostly medium to large in magnitude, with the exception of the AIM-A relationships/communication global domain. The results for this domain may have been impacted or skewed by eligibility criteria requiring stable relationships at study enrollment (all participants were required to have a reliable informant or significant other). Also, the relatively short study duration may have impacted the change scores and hence the effect size for the AIM-A relationships/communication global domain. For the AIM-A, all 6 domains were above the prespecified cut-off threshold for hierarchical testing; changes were all statistically significant, with effect sizes ranging from 0.31 to 0.93. The safety profile of LDX was consistent with long-acting psychostimulant use and other LDX studies.

## Abbreviations

AAQoL: Adult ADHD QoL; ADHD: Attention-deficit/hyperactivity disorder; ADHD-RS-IV: ADHD Rating scale version IV; AIM-A: Adult ADHD Impact module; ANCOVA: Analysis of covariance; BADDS: Brown attention-deficit disorder scale; BRIEF-A: Behavior rating inventory of EF-Adult; CGI-I: Clinical global impressions-improvement; CGI-S: Clinical global impressions-severity; DBP: Diastolic blood pressure; DSM-IV-TR: Diagnostic and statistical manual, fourth edition, text revision; ECG: Electrocardiogram; EF: Executive function; EFD: Executive function deficits; ES: Effect size; ET: Early termination; FAS: Full analysis set; GEC: Global executive composite; LDX: Lisdexamfetamine dimesylate; LS: Least squares; QOL: Quality of life; SBP: Systolic blood pressure; SD: Standard deviation; SE: Standard error; TEAEs: Treatment-emergent adverse events.

## Competing interests

**Lenard A**. **Adler**, **MD**, has been a consultant to AstraZeneca, Eli Lilly, Epi-Q, i3 Research, INC Research, Mindsite, Organon/Schering-Plough/Merck, Ortho-McNeil/Janssen/Johnson & Johnson, Otsuka, Shire, United Biosource, Major League Baseball, Major League Baseball Players Association, the National Football League, Alcobra Pharmaceuticals and Theravance; he has received research support from Bristol-Myers Squibb, Chelsea Therapeutics, Eli Lilly, Organon/Schering-Plough/Merck, Ortho-McNeil/Janssen/Johnson & Johnson and the National Institute of Drug Abuse (NIDA); he has been on the advisory boards of Eli Lilly, i3 Research, INC Research, Mindsite, Organon/Schering-Plough/Merck, Ortho-McNeil/Janssen/Johnson & Johnson, Theravance and Alcobra Pharmaceuticals. He has previously been (and has not been for the last three years) on the Speaker’s Board for Ortho-McNeil/Janssen/Johnson & Johnson, Shire and Eli Lilly. He has received an options grant from Alcobra Pharmaceuticals and has received royalty payments (as inventor) from New York University for license of adult ADHD scales and training materials. **Bryan Dirks**, **MD**, is an employee of Shire and holds stocks and/or stock options in Johnson & Johnson and Shire. **Patrick Deas**, **BS**, is an employee of Shire and holds stocks and/or stock options in Shire. **Aparna Raychaudhuri**, **PhD**, is an employee of Shire and holds stocks and/or stock options in Shire. **Matthew Dauphin**, **MS**, is an employee of Shire and holds stocks and/or stock options in Shire. **Keith Saylor**, **PhD**, has received research support from Shire, Eli Lilly, Otsuka, Supernus, Bristol-Myers Squibb, Novartis, Merck; is/has been a consultant or on an advisory board of Supernus Pharmaceuticals, Eli Lilly and Company, Shire. **Richard Weisler**, **MD**, in his career, has been a consultant to, on the Speaker’s Bureaus of, and/or received research support from the following: Abbott - Speaker’s Bureau, Consultant, Received Research Support, Agency for Toxic Substances and Disease Registry- Consultant, AstraZeneca - Speaker’s Bureau, Consultant, Received Research Support, Biovail - Speaker’s Bureau, Consultant, Received Research Support, Bristol-Myers Squibb - Speaker’s Bureau, Consultant, Received Research Support, Stockholder has held or holds stock, Burroughs Wellcome - Speaker’s Bureau, Received Research Support, Cenerx - Received Research Support, Centers for Disease Control and Prevention - Consultant, Cephalon - Speaker’s Bureau, Consultant, Received Research Support, Ciba Geigy - Speaker’s Bureau, Received Research Support, CoMentis - Received Research Support, Corcept - Consultant, Cortex - Stockholder has held or holds stock, Dainippon Sumitomo Pharma America - Received Research Support, Eisai - Received Research Support, Elan - Received Research Support, Eli Lilly - Speaker’s Bureau, Consultant, Received Research Support, Forest - Speaker’s Bureau, Consultant, Received Research Support, GlaxoSmithKline - Speaker’s Bureau, Consultant, Received Research Support, Janssen - Speaker’s Bureau, Received Research Support, Johnson & Johnson - Speaker’s Bureau, Consultant, Received Research Support, Lundbeck - Received Research Support, McNeil Pharmaceuticals - Received Research Support, Medicinova - Received Research Support, Medscape Advisory Board - Consultant, Merck - Received Research Support, Stockholder has held or holds stock, National Institute of Mental Health - Consultant, Received Research Support, Neurochem - Received Research Support, New River Pharmaceuticals - Received Research Support, Novartis - Speaker’s Bureau, Received Research Support, Organon - Speaker’s Bureau, Consultant, Received Research Support, Otsuka America Pharma - Consultant, Pfizer - Speaker’s Bureau, Consultant, Received Research Support, Stockholder has held or holds stock, Pharmacia - Consultant, Received Research Support, Repligen - Received Research Support, Saegis - Received Research Support, Sandoz - Received Research Support, Sanofi - Speaker’s Bureau, Consultant, Received Research Support, Sanofi-Synthelabo - Speaker’s Bureau, Consultant, Received Research Support, Schwabe/Ingenix - Received Research Support, Sepracor - Received Research Support, Shire - Speaker’s Bureau, Consultant, Received Research Support, Solvay - Speaker’s Bureau, Consultant, Sunovion - Speaker’s Bureau, Consultant, Received Research Support, Synaptic - Received Research Support, Takeda - Received Research Support, TAP - Received Research Support, Theravance - Received Research Support, Transcept Pharma - Consultant, Received Research Support, TransTech - Consultant, UCB Pharma - Received Research Support, Validus - Speaker’s Bureau, Consultant, Vela - Received Research Support, and Wyeth - Speaker’s Bureau, Consultant, Received Research Support.

## Authors’ contributions

LA was the principal investigator on the parent study and participated in data acquisition, analysis, interpretation, and presentation. LA was fully involved in drafting the manuscript and revising the intellectual content of this manuscript. BD was the Director, Clinical Development/Medical Affairs for this study and made substantial contributions to the analysis and interpretation of the data. He was deeply involved in drafting the manuscript and revising the intellectual content. PD was the Senior Clinical Scientist for this study and made substantial contributions to the analysis and interpretation of the data. He was deeply involved in drafting the manuscript and revising the intellectual content. AR was the statistician involved in all analysis, interpretation and presentation. She was fully involved in drafting and revising the intellectual content of this manuscript. MD was the Associate Clinical Programs Director for this study and made substantial contributions to the analysis and interpretation of the data. He was deeply involved in drafting the manuscript and revising the intellectual content. KS was an investigator on the parent study and participated in data acquisition, analysis, interpretation, and presentation. KS was fully involved in drafting the manuscript and revising the intellectual content of this manuscript. RW was an investigator on the parent study and participated in data acquisition, analysis, interpretation, and presentation. RW was fully involved in drafting the manuscript and revising the intellectual content of this manuscript. All authors read and approved the final manuscript.

## Pre-publication history

The pre-publication history for this paper can be accessed here:

http://www.biomedcentral.com/1471-244X/13/253/prepub
